# Anti-Sintering Behavior of GYYSZ, Thermophysical Properties, and Thermal Shock Behavior of Thermal Barrier Coating with YSZ/Composite/GYYSZ System by Atmospheric Plasma Spraying

**DOI:** 10.3390/nano14221787

**Published:** 2024-11-07

**Authors:** Chunxia Jiang, Rongbin Li, Feng He, Zhijun Cheng, Wenge Li, Yuantao Zhao

**Affiliations:** 1 School of Materials Science and Engineering, Shanghai Dianji University, Shanghai 201306, China; jiangcx@sdju.edu.cn (C.J.); haggsicff@163.com (F.H.); 181002500112@sdju.edu.cn (Z.C.); 2 Merchant Marine College, Shanghai Maritime University, Shanghai 201306, China; wgli@shmtu.edu.cn (W.L.); zhaoyt@shmtu.edu.cn (Y.Z.)

**Keywords:** thermal barrier coating, GYYSZ, atmospheric plasma spraying, phase stability, anti-sintering behavior, composite gradient coating, thermal shock resistance

## Abstract

In this study, Gd_2_O_3_ and Yb_2_O_3_ co-doped YSZ (GYYSZ) ceramic coatings were prepared via atmospheric plasma spraying (APS). The GYYSZ ceramic coatings were subjected to heat treatment at different temperatures for 5 h to analyze their high-temperature phase stability and sintering resistance. The thermophysical properties of GYYSZ, YSZ, and composite coatings were compared. Three types of thermal barrier coatings (TBCs) were designed: GYYSZ (TBC-1), YSZ/GYYSZ (TBC-2), and YSZ/Composite/GYYSZ (TBC-3). The failure mechanisms of these three TBCs were investigated. The results indicate that both the powder and the sprayed GYYSZ primarily maintain a homogeneous cubic phase c-ZrO_2_, remaining stable at 1500 °C after annealing. The sintering and densification of the coatings are influenced by the annealing temperature; higher temperatures lead to faster sintering rates. At 1500 °C, the grain size and porosity of GYYSZ are 4.66 μm and 9.9%, respectively. At 1000 °C, the thermal conductivity of GYYSZ is 1.35 W·m^−1^ K^−1^, which is 44% lower than that of YSZ. The thermal conductivity of the composite material remains between 1.79 W·m^−1^ K^−1^ and 1.99 W·m^−1^ K^−1^ from room temperature to 1000 °C, positioned between GYYSZ and YSZ. In the TBC thermal shock water quenching experiment, TBC-3 demonstrated an exceptionally long thermal shock lifetime of 246.3 cycles, which is 5.8 times that of TBC-1 and 1.8 times that of TBC-2. The gradient coating structure effectively reduces the thermal mismatch stress between layers, while the dense surface microcracks provide a certain toughening effect. Failure analysis of the TBC reveals that TBC-3 exhibits a mixed failure mode characterized by both spallation and localized peeling. The ultimate failure was attributed to the propagation of transverse cracks during the final stage of water quenching, which led to the eventual spallation of the ceramic blocks.

## 1. Introduction

Gas turbines are the core power equipment in thermal energy systems, widely used in electricity generation, marine, locomotive, and transportation sectors [1]. Currently, the inlet gas temperature for G/H class gas turbine stage 1 blades has reached 1370−1450 °C, while J class turbines experience inlet temperatures as high as 1600 °C [2]. TBC technology involves depositing materials with low thermal conductivity and phase stability at a high temperature onto the surface of high-temperature alloys, protecting the substrate from thermal damage. YSZ is widely utilized in gas turbines and aero engines due to its excellent thermal cycling performance, high melting point, and low thermal conductivity [3,4]. However, with industrial advancements, the shortcomings of YSZ have become apparent; it easily undergoes phase transformations, severe sintering, and oxidation when operating above 1200 °C. This not only diminishes the heat-insulating property of the coating but also alters internal stresses, leading to eventual spallation [5,6,7,8]. To achieve higher thermal efficiency in gas turbines, traditional YSZ TBCs are increasingly inadequate for elevated temperature demands, necessitating the development of new thermal barrier coatings with superior performance for more complex environments.

Doping YSZ with rare earth oxides can significantly enhance its thermophysical properties [9,10]. Research has found that doping with materials such as CeO_2_, Yb_2_O_3_, Er_2_O_3_, and Gd_2_O_3_ could reduce the YSZ coating’s thermal conductivity effectively. The reason for this is the differences in ionic radii between the dopants and Zr^4+^, which generate a greater number of oxygen vacancies and an increased concentration of lattice defects. Such changes enhance phonon scattering, thereby lowering thermal conductivity [11,12]. Partial substitution of Zr^4+^ with rare earth or alkaline earth oxides forms a substituted solid solution, which helps stabilize the tetragonal phase of ZrO_2_, allowing the material to maintain phase stability [13,14]. Because of the variations in mass and ionic radius between rare earth oxides and Zr^4+^ or Y^3+^, they can create a multi-point dislocation-complexing effect. This not only enhances the mechanical properties, sintering resistance, and stability of the materials but also improves the thermal cycling lifetimes of TBCs [15,16]. Chen [17] and Lei [18] prepared Yb_2_O_3_-Gd_2_O_3_ co-doped YSZ coatings of varying thicknesses using the APS method. Incorporating rare earth oxides can form an impermeable reaction layer, enhancing the thermal stability and corrosion resistance of YSZ, with multiple doping effects proving to be more effective [19,20,21]. Chen’s investigation of LGYYSZ revealed that the increased number of oxygen vacancies and defects, along with the mismatch in mass and size between these defects and the matrix lattice, diminished the average phonon mean free path. Consequently, the thermal conductivity of LGYYSZ ceramics at 1000 °C was recorded at 1.21 W·m^−1^·K^−1^, which is roughly 40% lower than that of YSZ. YbGdZrO ceramics sintered for 400 h at 1723 K exhibited no new phase formation and demonstrated excellent thermal stability [15]. The arithmetic average of the thermal diffusivity for YbGdZrO bulk at 1273 K was found to be 0.385 mm²/s, which is comparable to that of Gd_2_Zr_2_O_7_ ceramics (0.388 mm^2^/s at 1273 K) [22].

Nevertheless, these materials frequently demonstrate relatively low thermal expansion coefficients and inadequate mechanical properties. To address these shortcomings, extensive research and improvements have been made regarding TBCs in recent years. In addition to the typical bilayer thermal barrier coating structure, studies have also explored bilayer ceramic structures and functionally graded thermal barrier coatings. Bilayer ceramic coatings not only enhance the working temperature of the coatings but also extend their service life. Wang [23] used APS to prepare YSZ bilayer coatings and La_2_Zr_2_O_7_/8YSZ bilayer ceramic coatings. Experimental results showed that in the 1000 °C water quenching thermal cycling test, the bilayer structure failed at 46 times, while the bilayer ceramic thermal barrier coating reached 158 cycles. In the 1200 °C water quenching thermal cycling test, the thermal shock lifetimes were 7 and 43 cycles, respectively, significantly enhancing thermal shock performance. Xu [24] employed the EB-PVD to fabricate La_2_Zr_2_O_7_/8YSZ bilayer ceramic coatings, investigating their cyclic oxidation behavior. To further improve the interfacial bonding among the components, functionally graded thermal barrier coatings (FGTBCs) were designed. Numerical simulations have indicated that FGTBCs exhibit significantly enhanced thermal shock lifetimes [25,26]. Portinha [27] designed a porosity gradient structure, achieving over 100 cycles in thermal shock tests. Chen [28] conducted performance studies on La_2_Zr_2_O_7_/8YSZ, finding that the gradient coatings significantly outperformed traditional bilayer structures in thermal shock resistance. Additionally, Chen [29] developed an LMA/YSZ functionally graded TBC system. Experimental results indicated that at 1350 °C, the gradient coating exhibited a more durable thermal shock lifetime. Although the introduction of gradient structures has improved the thermal shock resistance and extended the service life of TBCs, the complexity of the gradient structure fabrication process poses challenges in accurately controlling the gradient profiles. Thus, there remains significant room for development in the research of gradient structure thermal barrier coatings.

GYYSZ is a promising new TBC material. Research on GYYSZ gradient thermal barrier coatings is still limited. This study employed APS technology to fabricate GYYSZ ceramic coatings, investigating their high-temperature phase stability and sintering resistance at various annealing temperatures. Bulk ceramics were produced through hot pressing, and the thermophysical properties, including the thermal conductivity and thermal expansion coefficients of GYYSZ, YSZ, and composite materials, were tested and evaluated. Three TBC structures were designed and fabricated: bilayer structures, bilayer ceramic structures, and functionally graded structures. The thermal shock resistance of these three TBCs was assessed through water quenching thermal shock experiments, exploring the failure mechanisms of each structure from the perspective of crack development.

## 2. Materials and Methods

**Figure 1:**
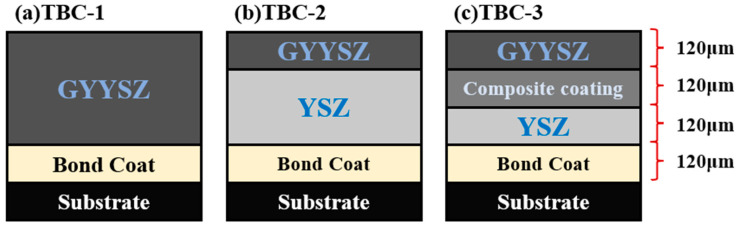
The schematic diagrams of the TBC structures: (**a**) bilayer; (**b**) bilayer ceramic; (**c**) functionally graded ceramic.

The thermal barrier coating substrate material selected for this experiment is the 718 nickel alloy. Its dimensions are Ф 25 mm × 5 mm. The sprayed powder has a micrometer-scale particle size. The bonding layer of NiCrAlY uses commercial powder Amdry 962 from Oerlikon Metco, Vista, NSW, Australia, with the composition of Ni-22Cr-10Al-1.0Y. The ceramic layers consist of YSZ (ZrO_2_-8Y_2_O_3_, Metco 204NS, Oerlikon Metco) and GYYSZ (ZrO_2_-9.5Y_2_O_3_-5.6Yb_2_O_3_-5.2Gd_2_O_3_, Metco 206A, Oerlikon Metco). The composite coating is prepared by mixing YSZ and GYYSZ powders in a mass ratio of 1:1, with the composite powder created through ball milling prior to spraying. To investigate the high-temperature phase stability and sintering resistance of GYYSZ, coatings were sprayed onto 304 stainless steel and subjected to multiple water quenching processes to obtain independent ceramic layers. For assessing the thermophysical properties of the coating materials, ceramic bulk samples of YSZ, GYYSZ, and composite structures were fabricated using ball milling and hot pressing, with dimensions of Ф 12.7 × 1 mm and Ф 6 × 25 mm. The coatings were prepared using the Multicoat System plasma spraying system from Oerlikon Metco. Due to the varying characteristics of different powders, experimental parameters listed in Table 1 were selected for this study after consulting the relevant literature and conducting multiple experimental adjustments [30,31]. The designs of the three TBC structures are illustrated in Figure 1. The number of samples sprayed using the same spraying process is 9.

Phase analysis was conducted using a BRUKER D8 X-ray diffractometer (XRD) with Cu target Kα radiation (λ = 1.5418 Å) at 40 kV and 40 mA. The 2θ scanning range was set from 20° to 90°, with a scanning speed of 5°/min and a step size of 0.02°. The surface and cross-sectional morphology of the coatings, as well as the elemental composition, were observed using a Zeiss GeminiSEM 300 field emission scanning electron microscope (SEM) (Zeiss, Jena, Germany) and a HITACHI S-3400N tungsten filament SEM (Hitachi, Tokyo, Japan), equipped with an energy dispersive X-ray spectrometer (EDS). In the BSE mode of the SEMs, 10 random positions were selected at 500× magnification to observe morphology, and the images were processed using ImageJ software (ImageJ 1.54i) to calculate the porosity of the samples. Ten points were chosen for statistical analysis, with the average value representing the final porosity of the coating. Within ImageJ, five random lines were drawn on SEM grain images at 5000× magnification to measure the true lengths and count the grains intercepted by the lines, allowing for the calculation of average grain size. Thermal conductivity and thermal expansion coefficients of the coating materials were tested using a Netzsch LFA 457 laser flash apparatus and a DIL 402CL dilatometer (Netzsch, Selb, Germany).

Samples were heat-treated using a Nabertherm N61/H atmosphere furnace (Nabertherm, Lilienthal, Germany). The thermal shock testing method for the TBCs involved photographing and weighing TBC-1, TBC-2, and TBC-3 before the experiment. Once the furnace temperature reached 1150 °C, the furnace door was quickly opened to insert the samples, which were held for 5 min. The samples were then rapidly removed and plunged into 25 °C deionized water for quick cooling, soaking for 2 min. After drying, the samples were photographed and weighed again, completing one cycle. This procedure was repeated until a 20% coating delamination was observed, defining the number of cycles at which this occurred as the thermal shock life. Three samples were taken for each set of TBC parameters to undergo thermal shock and water quenching tests. and the average value calculated from these tests represented the average thermal shock life.

## 3. Results and Discussion

### 3.1. Microstructural and Property Analysis of GYYSZ Coating

**Figure 2:**
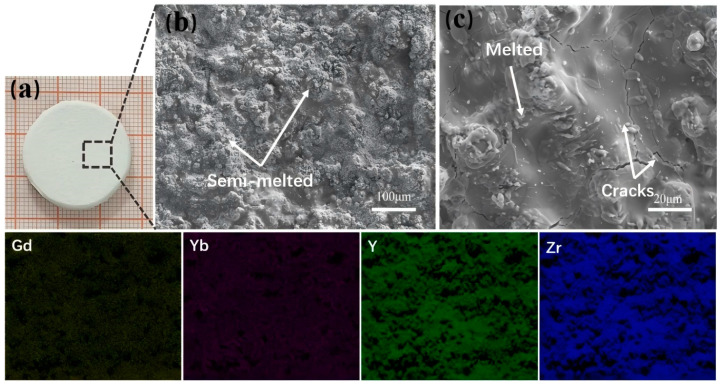
(**a**) The GYYSZ ceramic coating macroscopic photograph; (**b**,**c**) the surface morphology; and the EDS mapping.

Figure 2 presents the macroscopic photograph, surface morphology, and the EDS elemental distribution map of the sprayed GYYSZ ceramic coating. In Figure 2a, the coating surface appears to be smooth and milky white, matching the color of the ceramic powder. Figure 2b shows no obvious spherical structure of the GYYSZ ceramic powder, indicating that the particles melted in the high-temperature environment of APS. The magnified view reveals two distinct areas on the coating surface: a smooth, fully molten region and a semi-molten region where ceramic powder remains partially unmelted. The height difference between the semi-molten and fully molten regions is due to the varying kinetic energy of the powder particles as they are propelled by the high-speed flame flowing from the nozzle. This variation affects their final state upon impact with the substrate or coating. Ceramic powders with a higher degree of melting exhibit liquid characteristics upon impact, causing them to splash and spread out, forming a layered coating. This type of coating is fully molten and demonstrates good density and adhesion to the substrate. However, during the deposition of these coatings, quenching stresses can occur, leading to the formation of microcracks, as illustrated in Figure 2c. In contrast, ceramic powders with a lower degree of melting tend to bond and stack upon impact with the substrate or may fall off, resulting in increased porosity in the coating. The EDS elemental mapping conducted at the position indicated in Figure 2b reveals that the elements Gd, Yb, Y, and Zr are uniformly distributed throughout the GYYSZ ceramic coating.

**Figure 3:**
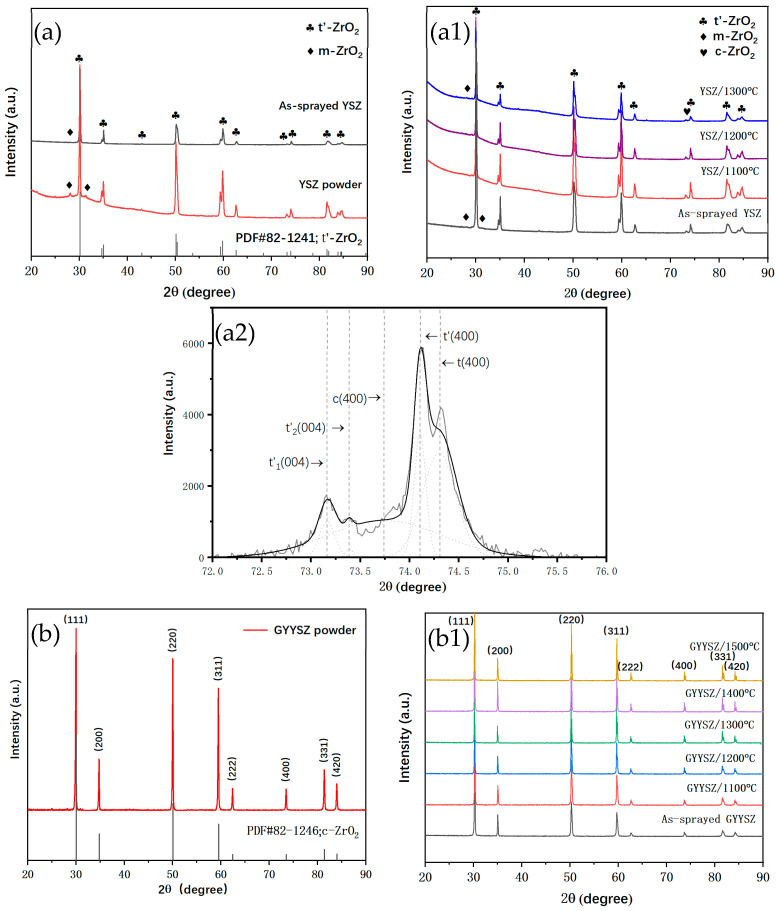
(**a**) The XRD patterns of the YSZ powder and coating; (**a1**) the XRD patterns of the YSZ ceramic coating after heat treatment for 5 h at different temperatures; (**a2**) the XRD patterns and fitting curves in the 2θ = 72.5°–75° range of the YSZ coating after heat treatment at 1300 °C; (**b**) the XRD patterns of GYYSZ in the sprayed state and powder state; (**b1**) the XRD patterns of the GYYSZ ceramic coating after heat treatment for 5 h at different temperatures.

Figure 3 presents the XRD patterns of YSZ and GYYSZ in their powdered forms, their as-sprayed states, and following heat treatment at various temperatures. It can be seen from Figure 3a that the YSZ ceramic powder and coating primarily consist of the metastable tetragonal phase t′-ZrO_2_, with the powder state containing a significant amount of monoclinic phase m-ZrO_2_ [17]. In the sprayed state, the intensity of the m-ZrO_2_ characteristic peaks is reduced due to the phase transformation of the ceramic powder at the high temperatures of APS, leading to the formation of t′-ZrO_2_. The rapid cooling after spraying restricts the phase transformation from t′ to t to m. Figure 3(a1) displays the XRD patterns of the YSZ coating following heat treatment at various temperatures. After treatment at 1100 °C, the m-ZrO_2_ phase in the sprayed YSZ coating gradually disappears. However, after heat treatment, a new phase of Y_2_O_3_ appears at approximately 2θ ≈ 29°, indicating that some of the stabilizer Y_2_O_3_ precipitates from the YSZ above 1100 °C. After heat treatment at 1300 °C, the monoclinic phase m-ZrO_2_ reappears at approximately 2θ ≈ 28°, suggesting that a partial phase transformation of the t′ phase in YSZ has occurred. The phase transformation of t′-ZrO_2_ at high temperatures results in the formation of a three-phase mixture, which includes a Y-poor tetragonal phase ZrO_2_ [32], a Y-rich cubic phase c-ZrO_2_, and a tetragonal phase ZrO_2_. Among them, the Y-poor tetragonal phase ZrO_2_ has a higher tetragonality and is less able to maintain its metastable structure, gradually transforming into the monoclinic phase as the temperature decreases. The Y-rich c-ZrO_2_ is retained, which explains the presence of the diffraction peak for c-ZrO_2_ at approximately 2θ ≈ 74° in the XRD pattern of YSZ after heat treatment at 1300 °C. To assess the extent of phase transformation in YSZ after heat treatment at 1300 °C, the XRD patterns in the range of 2θ = 72.5°–75° were analyzed, as shown in Figure 3(a2). In this range of diffraction angles, the ZrO_2_ lattice exhibits only the tetragonal and cubic phases. The t′-ZrO_2_ features characteristic peaks at (004) and (400). In this analysis, curve fitting of the diffraction patterns allows for the determination of peak integral intensities, which can then be used to calculate the molar ratio of cubic ZrO_2_ to tetragonal ZrO_2_. By combining the integral intensities of the monoclinic and tetragonal phases in the range of 2θ = 27.5°–32°, the contents of each ZrO_2_ phase in YSZ after heat treatment at 1300 °C can be calculated using Formulas (1)–(3).

(1) $$\frac{M_{m}}{M_{c}{+M}_{t}}=0.82(\frac{I_{{\left(\bar{1}11\right)}_{m}}+I_{{\left(111\right)}_{m}}}{I_{{\left(111\right)}_{c,t}}})$$

(2) $$\frac{M_{c}}{M_{t}}=0.88(\frac{I_{{\left(400\right)}_{c}}}{I_{{\left(004\right)}_{t}}+I_{{\left(400\right)}_{t}}})$$

(3) $$M_{m}+{M_{c}+M}_{t}=1$$

M_m_, M_c_, and M_t_ represent the molar fractions of the monoclinic, cubic, and tetragonal phases, respectively, while I denotes the integral intensities of the corresponding characteristic peaks. After calculations, the contents of cubic c-ZrO_2_ and tetragonal t-ZrO_2_ in YSZ treated at 1300 °C were found to be 9.12% and 18.52%, respectively.

Figure 3b shows the XRD patterns of GYYSZ in powder form, in its sprayed state, and after heat treatment at different temperatures. The diffraction peak angles of the sprayed GYYSZ shifted approximately 0.1° towards higher angles, indicating that the GYYSZ coating has a smaller lattice constant compared to the powder form.

(4) 2dsinθ=nλ

The refractive index *n* and wavelength *λ* are constant, and the diffraction angle *θ* increases as the interplanar spacing *d* decreases. GYYSZ involves the substitution of Zr sites in the ZrO_2_ lattice by Y^3+^, Gd^3+^, and Yb^3+^ ions. The ionic radii of Y^3+^, Gd^3+^, Yb^3+^, and Zr^4+^ are shown in Table 2, where r(Gd^3+^) > r(Y^3+^) > r(Yb^3+^) > r(Zr^4+^). Doping ZrO_2_ with these three rare earth cations leads to lattice distortion, resulting in an increase in interplanar spacing. At approximately 2θ ≈ 29.2°, a weak Y_2_O_3_ diffraction peak appears, indicating the presence of trace Y_2_O_3_ precipitation. This further confirms that the overall shift to higher angles in the sprayed state is due to the precipitation of rare earth cations during the spraying process. Figure 3(b1) shows the XRD patterns of the coating after heat treatment. After treatment at 1500 °C for 5 h, the phase structure of GYYSZ remains consistent with the sprayed state, which is the c-ZrO_2_. This indicates that GYYSZ exhibits better high-temperature structural stability compared to YSZ materials.

**Figure 4:**
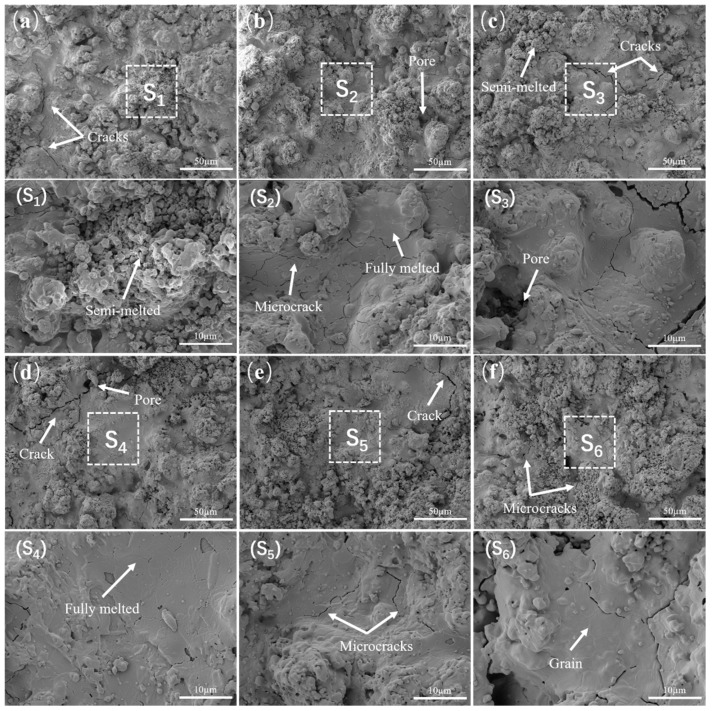
The surface morphology images of GYYSZ coatings after heat treatment for 5 h: (**a**) as-sprayed; (**b**) 1100 °C; (**c**) 1200 °C; (**d**) 1300 °C; (**e**) 1400 °C; and (**f**) 1500 °C.

Figure 4 shows the surface morphology images of GYYSZ coatings after heat treatment at different temperatures. As illustrated, the surface of the GYYSZ coating distinctly exhibits a distribution of semi-melted ceramic powder particles and fully melted ceramic layers, which is evident throughout the entire heat treatment process. The semi-melted region is rough and distinctly granular, with a scattering of various-sized pores. In contrast, the fully melted region is smooth and even, featuring a certain number of microcracks. These microcracks are a result of residual stresses induced during the cooling process after spraying. Due to the relatively low fracture toughness of GYYSZ ceramics, microcracks can initiate in the coating under the influence of residual stress. The unmelted nanoparticles in the coating are shown in Figure 4(S1). The nanoparticles exhibit a regular shape, mostly consisting of irregularly sized polyhedra. After heat treatment at different temperatures for 5 h, the shapes of the nanoparticles are depicted in Figure 4(S2–S6), where the edges of the nanoparticles gradually become rounded, and the bonding rate between the layers increases. Comparing the sintering behavior of GYYSZ coatings at various temperatures ranging from 1100 °C to 1500 °C, the sintering process gradually intensifies. Above 1300 °C, some microcracks progressively disappear during sintering.

**Figure 5:**
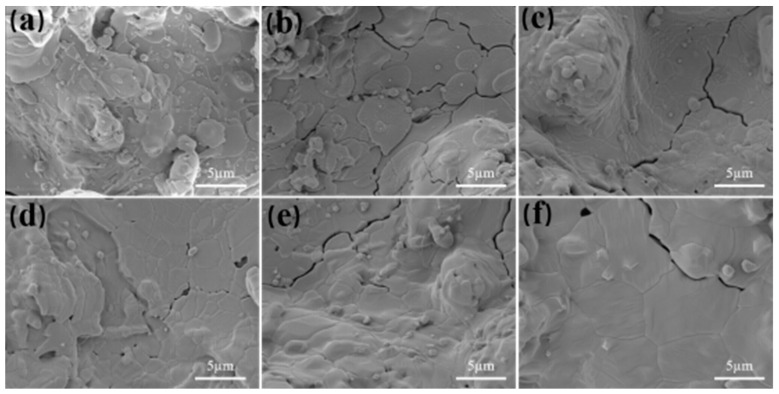
The grain distributions of GYYSZ coatings after heat treatment for 5 h: (**a**) as-sprayed; (**b**) 1100 °C; (**c**) 1200 °C; (**d**) 1300 °C; (**e**) 1400 °C; (**f**) 1500 °C.

Figure 5 illustrates the grain distribution of GYYSZ coatings following heat treatment at various temperatures. In the as-sprayed state, no visible grains are present in the GYYSZ coating. Slight sintering occurs, and grains can be observed to be bonding to each other, after heat treatment at 1100 °C. The grain size grows, the grain texture becomes clearer, and the number of microcracks diminishes with increasing heat treatment temperature. Table 3 presents the grain sizes of GYYSZ coatings following heat treatment at various temperatures. At 1100 °C, the coating begins to sinter, and the average grain size is 840 nm. As the temperature increases, the grain size gradually enlarges, and at 1500 °C, significant sintering occurs, with the average grain size reaching 4657 nm, which is 5.5 times the grain size at 1100 °C.

**Figure 6:**
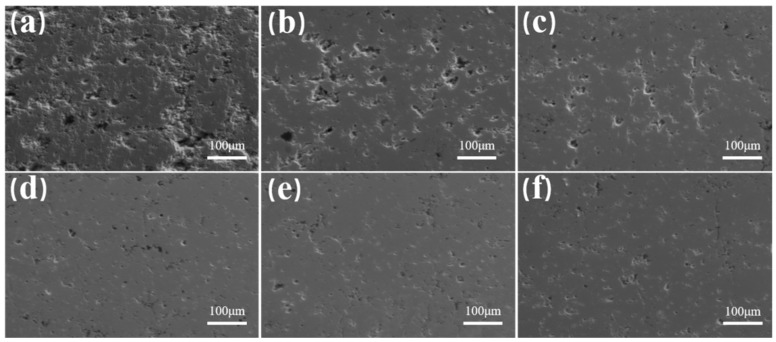
The cross-sectional morphology of GYYSZ coatings after heat treatment at different temperatures: (**a**) as-sprayed; (**b**) 1100 °C; (**c**) 1200 °C; (**d**) 1300 °C; (**e**) 1400 °C; (**f**) 1500 °C.

Figure 6 depicts the cross-sectional images of GYYSZ coatings following heat treatment at various temperatures. As seen in Figure 6a, the high-temperature sintered coatings exhibit significant porosity. In the cross-section, pores combine with microcracks, forming extensive porous regions. The interior of the pores has a rough texture, with some partially unmelted ceramic powder particles present. These pores result from the stacking of incompletely melted powder during the spraying process. The number of microcracks is relatively reduced, and the coating exhibits slight sintering, but the pore diameter has not significantly changed, in Figure 6b. This indicates that, in the initial stage of sintering, the contact area between particles increases. As the heat treatment temperature rises, the pore diameter decreases, the roundness of the pores improves, and the irregular pores gradually develop into holes. At 1300 °C, the porosity of the coating significantly decreases, and the degree of densification increases, indicating that the densification has entered a quasi-steady state. In the final stage of sintering, the large pores in the coating begin to shrink until the holes close. At this stage, the sintering process is primarily driven by sintering stress caused by surface tension, and it lasts for a relatively long time [34]. Table 4 presents the cross-sectional porosity of GYYSZ coatings following heat treatment. As seen in the table, when the treatment temperatures are 1300 °C, 1400 °C, and 1500 °C, the porosities are 10.5%, 11.5%, and 9.9%, respectively. These porosity values are lower than those of the coatings after heat treatment at 1100 °C and 1200 °C under the same annealing time, indicating that higher sintering temperatures accelerate the densification process of the coatings [35]. The porosity no longer decreases at 1300 °C, suggesting that the GYYSZ coating has entered a quasi-steady state of densification. A certain level of porosity is beneficial for reducing phonon-mediated heat transfer, thereby lowering the thermal conductivity of the coating. The fact that the coating maintains a certain porosity even at 1500 °C indicates that GYYSZ has good sintering resistance and maintains structural stability at elevated temperatures.

### 3.2. Design and Performance Study of Gradient Thermal Barrier Coatings

As noted in Section 3.1, GYYSZ exhibits excellent high-temperature anti-sintering capability and high-temperature phase stability, making it a thermal barrier coating material with great development potential. However, the considerable difference in thermal expansion coefficients in GYYSZ and the bonding layer can lead to significant thermal stresses developing within the coating during thermal cycling. When thermal stresses within the coating accumulate to a critical level, they can trigger the initiation and propagation of cracks, jeopardizing the lifespan of the coating. This study designed a composite coating consisting of a 1:1 weighted percentage mixture of YSZ and GYYSZ as an intermediate layer, facilitating thermal expansion transition and improving the thermal cycling durability of the coating.

**Figure 7:**
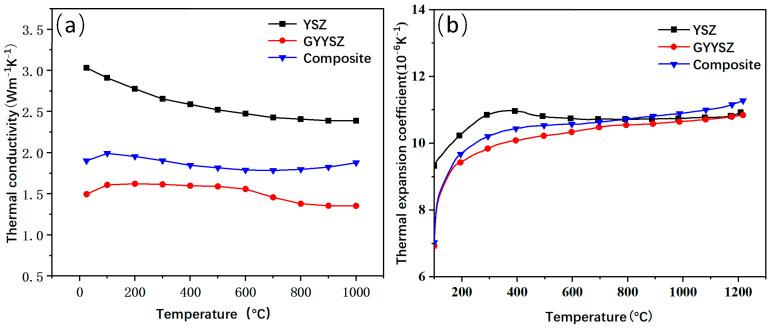
(**a**) The thermal conductivity; (**b**) the thermal expansion coefficient for YSZ [38], GYYSZ, and the composite material.

Figure 7 shows the curves of the thermal conductivity and the thermal expansion coefficient for YSZ, GYYSZ, and the composite material as a function of temperature. All three materials exhibit higher thermal conductivity at low temperatures and lower thermal conductivity at high temperatures, which aligns with the phonon scattering model. At elevated temperatures, increased phonon scattering reduces the average phonon mean free path, resulting in a decrease in the thermal conductivity of the samples. The thermal conductivity of YSZ is higher than that of GYYSZ and the composite material across the entire temperature range from room temperature to 1000 °C. YSZ’s thermal conductivity continuously decreases with increasing temperature, with a maximum value of 3.03 W·m^−1^ K^−1^ at room temperature and a minimum of 2.39 W·m^−1^ K^−1^ at 1000 °C. This is approximately 4/5 higher than the thermal conductivity of GYYSZ at the same temperature (1.35 W·m^−1^ K^−1^). The introduction of more dopant ions in GYYSZ creates a greater number of oxygen vacancies, which affects the average free path of phonons [36]. The thermal conductivity of the composite material falls between that of YSZ and GYYSZ. Similarly to GYYSZ, the thermal conductivity of the composite material follows a trend of initially increasing, then decreasing, and rising again as the temperature increases. At 200 °C, the thermal conductivity of the composite material reaches its maximum value of 1.99 W·m^−1^ K^−1^, and at 700 °C, it reaches its minimum value of 1.79 W·m^−1^ K^−1^. After that, the thermal conductivity gradually increases. This increase in thermal conductivity at higher temperatures is due to the densification of the sample at that temperature. At the same temperature, the minimum thermal conductivity of the composite material is still about 1/5 higher than that of GYYSZ. Overall, the thermal conductivity of the composite material remains between 1.79 W·m^−1^ K^−1^ and 1.99 W·m^−1^ K^−1^ from room temperature to 1000 °C, lying between the thermal conductivities of YSZ and GYYSZ. Figure 7b shows the curve of the thermal expansion coefficient for YSZ, GYYSZ, and the composite material as a function of temperature. In the temperature range of 100−1200 °C, the thermal expansion coefficient of the composite material increases with rising temperature. Below 300 °C, its value rapidly increases from 7.05 × 10^−6^ K^−1^ to 10.27 × 10^−6^ K^−1^. In contrast, the thermal expansion coefficient of YSZ increases only slightly in the 100−300 °C range, from 9.33 × 10^−6^ K^−1^ to 10.88 × 10^−6^ K^−1^. This indicates that YSZ has a slower rate of increase in its thermal expansion coefficient, leading to a more gradual growth in thermal strain. This more stable behavior during cooling helps to effectively reduce the generation of thermal stress, thereby lowering the likelihood of coating delamination and failure during thermal shock [37]. At temperatures ranging from 100 to 800 °C, the thermal expansion coefficients follow the order of YSZ > composite material > GYYSZ. At 800 °C, the thermal expansion coefficients of the composite and YSZ become similar, approximately 10.7 × 10^−6^ K^−1^. As the temperature increases, the thermal expansion coefficient of the composite increases in sync with GYYSZ. Therefore, the composite material can be used as an intermediate layer to bridge GYYSZ and YSZ, facilitating a transition in thermal expansion coefficients among the components of the thermal barrier coating and addressing the issue of thermal mismatch caused by the low thermal expansion coefficient of GYYSZ.

**Figure 8:**
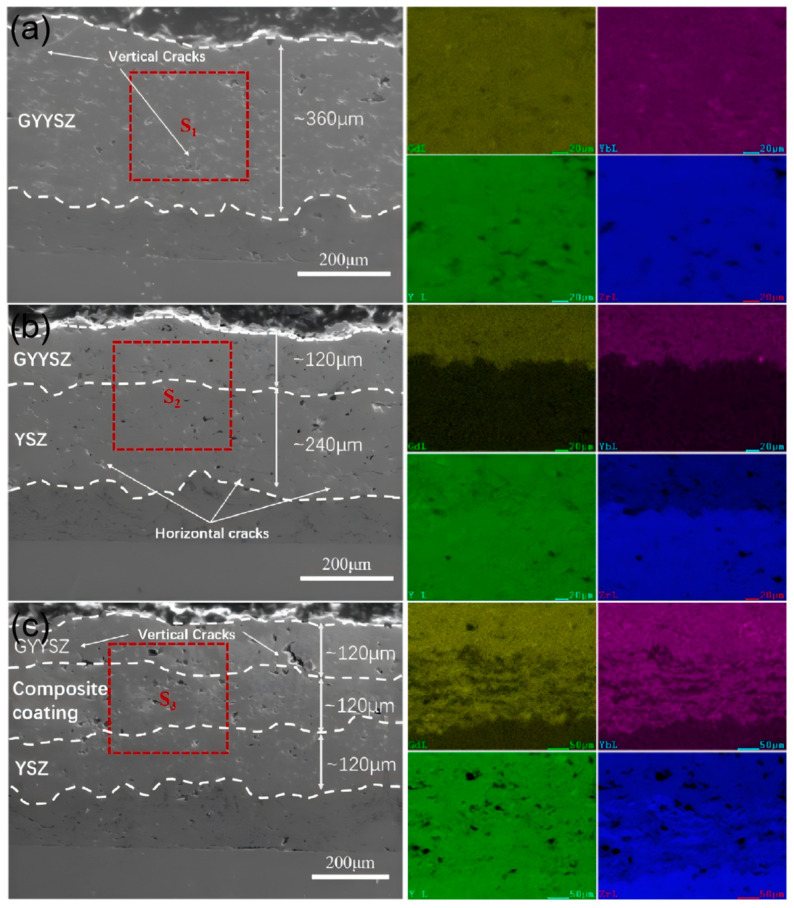
The cross-sectional SEM images and EDS element distribution maps for (**a**) TBC-1; (**b**) TBC-2; and (**c**) TBC-3.

Figure 8 shows the cross-sectional images of three different TBCs prepared using APS technology. GYYSZ is deposited on the top layer of the TBC. TBC-1 serves as the control group without a transition layer. In TBC-2 and TBC-3, YSZ acts as the transition layer. This is because YSZ has a relatively high thermal expansion coefficient and is deposited between the GYYSZ and the bonding layer (NiCrAlY) to establish a thermal expansion coefficient transition. In TBC-3, a composite coating is sprayed between the YSZ and GYYSZ layers, further enhancing the transition in thermal expansion coefficients. Figure 8a shows the cross-sectional microstructure and localized elemental distribution of TBC-1. The thickness of the GYYSZ layer is approximately 360 μm. The GYYSZ layer contains a significant number of pores and cracks, with the cracks predominantly oriented vertically. The porosity is about 20.97%. The metallic bonding layer and NiCrAlY are well-melted, and the interface between the ceramic layer and the bonding layer is distinct, with the boundary appearing as an irregular curve. This irregularity helps increase the contact area between the ceramic and bonding layers, thereby enhancing the bonding strength. Nevertheless, because of the significant porosity of the GYYSZ layer, pores are also present at the interface edges, which may affect the bonding strength. From the EDS elemental mapping results, it can be observed that the distribution of Gd, Yb, Y, and Zr elements is uniform. The GYYSZ top layer has a thickness of approximately 120 μm and the YSZ intermediate layer is around 240 μm thick in Figure 8b. The porosity of the as-sprayed YSZ layer is about 7.59%, and transverse microcracks are primarily distributed within the coating. The interface between the bonding layer and the ceramic layer is clearly defined, indicating good adhesion. However, it is difficult to distinguish the boundary between GYYSZ and YSZ in the SEM images due to their similar compositions and contrast. By performing an EDS elemental mapping on the selected region S2, the boundary between the coatings can be observed, showing that GYYSZ contains higher concentrations of Gd and Yb, while YSZ has a higher Zr content. Figure 8c shows the SEM image of the graded thermal barrier coating and the corresponding local EDS element distribution. It can be observed that the thickness of the three ceramic layers is approximately 120 μm. There are a certain number of pores in the cross-section of both the GYYSZ and composite layers, and the GYYSZ top layer exhibits some vertical cracks. EDS mapping of the S3 region reveals an alternating distribution of GYYSZ and YSZ within the composite layer. Additionally, the interfaces between the ceramic layers are clearly visible, with the boundary lines being irregular and winding, showing no significant cracks, indicating good bonding between the layers of the graded thermal barrier coating.

**Figure 9:**
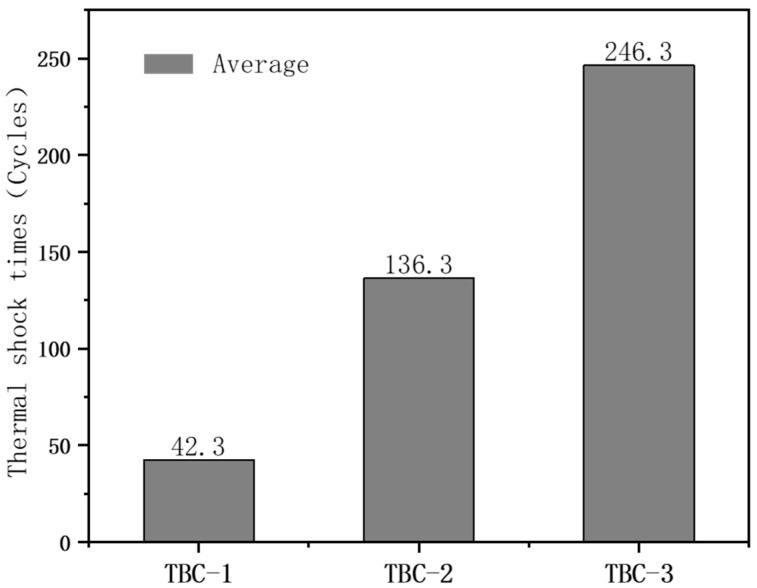
Average thermal shock times of three different structural TBCs.

Figure 9 illustrates the average thermal shock times of the three types of TBCs. The average thermal shock time is the shortest for TBC-1, at just 42.3 cycles. In contrast, TBC-2 shows a significant increase in thermal shock time, reaching an average of 136.3 cycles, which is 3.2 times that of TBC-1. This indicates that the dual-ceramic layer structure offers significantly better thermal shock resistance. Due to the low thermal expansion coefficient of GYYSZ, thermal mismatch occurs between the ceramic layer and the bonding layer in TBC-1. Depositing a layer of YSZ between GYYSZ and the bonding layer, the higher thermal expansion coefficient of YSZ at mid to low temperatures generates less thermal stress during thermal cycling compared to TBC-1, thereby extending the thermal cycling times of the coating. The average thermal shock time of TBC-3 is 246.3 cycles, nearly six times that of TBC-1 and 80% more than TBC-2. This indicates that adding a transition layer can significantly enhance the thermal shock lifespan of the TBC. The thermal shock test results show that modifying the compositional gradient of the TBC structure can enhance its thermal shock lifespan. Furthermore, adding a transition layer in coatings prone to thermal mismatch can create a gradient in thermal expansion coefficients, significantly improving thermal shock resistance.

**Figure 10:**
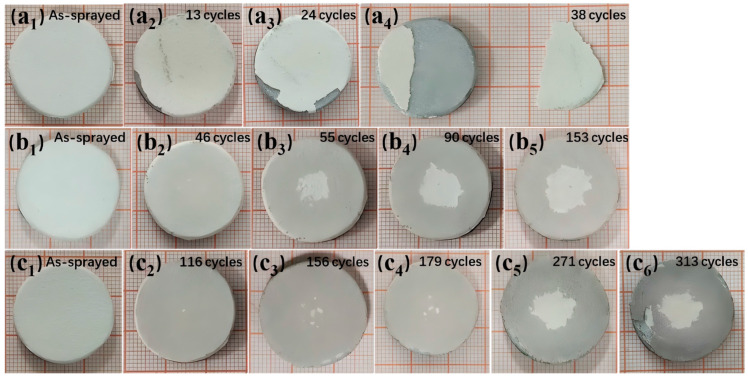
Macro photographs of three TBCs after varying times of thermal cycles: (**a1**–**a4**) TBC-1; (**b1**–**b5**) TBC-2; (**c1**–**c6**) TBC-3.

Figure 10 shows photographs of the three types of TBCs during thermal cycling experiments at different times of cycles, focusing on four stages: at the as-sprayed stage, at initial spalling, during ongoing spalling, and at thermal shock failure. The TBC failure mode is mainly in the form of ceramic layer peeling. TBC-1 exhibits large-scale spalling of the ceramic layer; TBC-2 shows failure in the center area with a failure area reaching 20%; TBC-3 demonstrates spalling failures at both the edges and the center of the ceramic layer. As shown in Figure 10(a1–a4), TBC-1 shows small spalling at the edges of the ceramic layer after 13 thermal shock cycles. As the number of cycles increases, the spalled area gradually enlarges, and by the 38th cycle, large areas of the ceramic layer have spalled off, displaying a gray color, leading to the termination of the experiment. When TBC-1 has completely failed, neither TBC-2 nor TBC-3 show any signs of ceramic layer spalling. However, after 46 thermal cycles, as shown in Figure 10(b2), visible spalling areas appear on the surface of TBC-2, with the rate of ceramic spalling increasing rapidly. Following an additional 9 water quenching cycles, the spalled area reaches 7.6% (Figure 10(b3)). Subsequently, the spalling rate decreases, and the spalled area slowly increases with the number of thermal shock cycles, ultimately failing after the 153rd cycle. Figure 10(c2–c6) show the spalling process of TBC-3. After 116 thermal quenching cycles, small spalling areas appear at the center of the coating, with the spalled area expanding slowly as the number of thermal cycles increases. After 156 thermal shock cycles, spalling is observed at the edges, but by the 179th thermal cycle, the spalled area still accounts for less than 1%. TBC-3 fails after 313 thermal shock cycles, and the failure modes of the two failure regions are similar to those of TBC-2.

**Figure 11:**
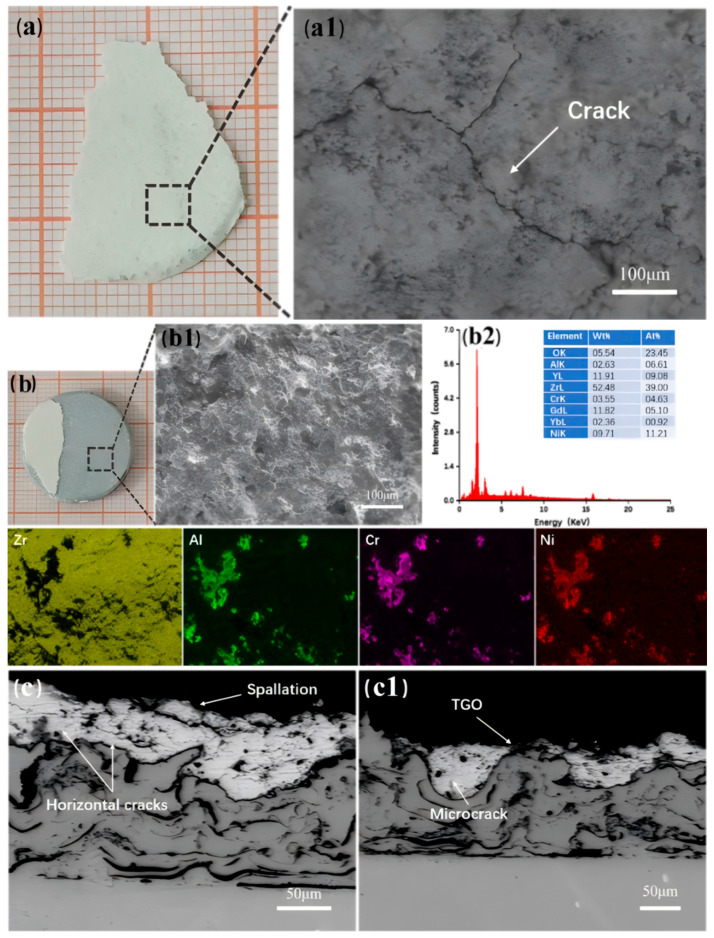
(**a**) The failure surface morphology of TBC-1; (**a1**) BSE diffraction image; (**b**) failure photographs of TBC-1; (**b1**) surface morphology of the failure location; (**b2**) EDS results and elemental distribution; and (**c**,**c1**) the cross-sectional microstructure of two locations of TBC-1 after thermal shock failure.

**Figure 12:**
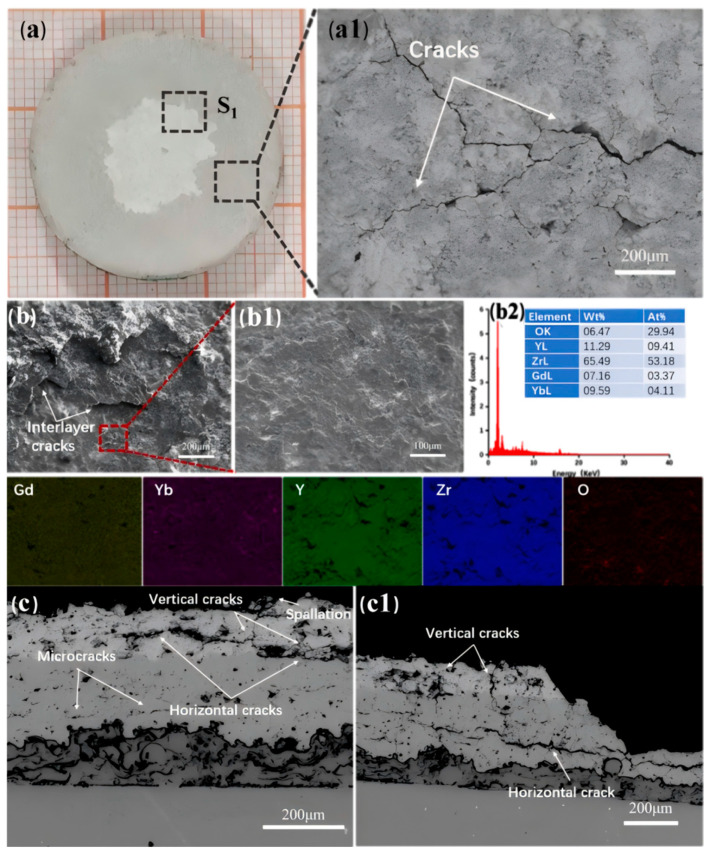
(**a**) Failure surface morphology of TBC-2; (**a1**) BSE image; (**b**) failure image of TBC-1; (**b1**) surface morphology of failure area; (**b2**) EDS mapping; (**c**,**c1**) cross-sectional microstructure of TBC-1 at two locations after thermal shock failure.

Figure 11 shows images of TBC-1 failure. As seen in Figure 11(a1), the surface of the fallen layer exhibits cracks. This occurs during heating, where the high thermal expansion coefficient of the bonding layer exerts compressive stress on the ceramic layer, while during cooling, the ceramic layer experiences tensile stress. In the thermal shock cycling experiment, the coating experiences a repetitive cycle of tensile and compressive stresses, which induces the initiation, and propagation of microcracks. After the ceramic layer of TBC-1 delaminates, the exposed area appears gray, indicating that the delamination occurs near the interface between GYYSZ and the bonding layer. The EDS elemental mapping results for the failed regions are shown in Figure 11(b1) and Figure 12(b2). The surface of the failed area is rough, and the EDS analysis indicates that, in addition to Gd, Yb, Zr, and Y, there is a significant presence of Ni, Cr, and Al elements in the areas where Zr is missing. Ni, Cr, and Al are the main components of the bonding layer. This suggests that the failure of the coating occurred at the interface between the ceramic layer and the bonding layer. Figure 11c shows the cross-sectional view of TBC-1 after failure. The coating surface has lost most of its ceramic layer due to thermal shock, as seen in Figure 11(c1). Although no through-thickness transverse cracks are observable in the area where the ceramic layer has detached, small transverse cracks are still visible. This is due to the significant thermal mismatch stress generated during thermal shock, caused by the large difference in thermal expansion coefficients between the ceramic layer and the bonding layer. This leads to the initiation of transverse cracks in the low-toughness ceramic, which propagate during thermal cycling [39,40]. TBC-1 has undergone few thermal shock cycles, so the accumulated stress in the ceramic layer has not yet reached the bonding strength between the ceramic materials. The competition between longitudinal cracks and lateral interface cracks is dominated by the development of lateral interface cracks, ultimately leading to the delamination observed in Figure 11(c1). The final failure of TBC-1 is attributed to the through-thickness transverse cracks caused by thermal mismatch stress.

Figure 12 shows the failure images of TBC-2. Figure 12(a1) displays the BSE photograph of the unspalled area of TBC-2. In the image, a network of surface cracks can be observed on the coating surface. When the thermal mismatch coefficients of the components in the TBC are significant, the ceramic layer experiences thermal mismatch stress. During cooling, the surface of the ceramic layer is subjected to substantial tensile stress, inducing cracks. These cracks initiate at the center of the sample and propagate downward vertically during the cyclical thermal cycling process. From Figure 12b, There is an obvious high and low difference on the coating [41]. The layer is peeling step by step, and obvious interlayer cracks can be observed, which is the result of transverse cracks and longitudinal cracks between the ceramic layer and peeling successively. An EDS mapping of the lowest ceramic layer indicated that this area is still GYYSZ. This suggests that the initial peeling occurred within the GYYSZ layer, followed by the formation of inter-layer cracks that converged with longitudinal cracks, resulting in localized peeling. Subsequent peeling events continued to occur within this layer. Figure 12c shows the cross-sectional structure of the unspalled area and the spalled area of TBC-2. In the unspalled area, both transverse and longitudinal cracks are present in the ceramic layer. The transverse cracks are mainly located at two interfaces: one is between GYYSZ and YSZ, and the other is between YSZ and NiCrAlY. The former has developed into through-type cracks, posing a risk of spalling at any moment, while the latter is still in the stage of expansion. There are two reasons for this situation: First, GYYSZ and YSZ tend to generate larger thermal mismatch stresses, leading to faster crack propagation at the GYYSZ/YSZ interface under the same number of thermal cycles. Second, due to the inherent properties of YSZ, when cracks are generated and propagate at the coating interface under tensile stress, the stress at the crack tip can induce a martensitic phase transformation from the t’ to the m phase in YSZ, primarily from the t’-ZrO_2_ phase. This phase transformation induces a volume expansion that partially offsets the tensile stress as compressive stress, enhancing toughness and effectively delaying the propagation of cracks at the YSZ/NiCrAlY interface. The addition of the YSZ layer replaces the direct contact between GYYSZ and the bonding layer, effectively alleviating interface stress, delaying coating spallation, and enhancing the lifespan of the coating. During the cooling process, the tensile stress on the surface of the GYYSZ coating increases with the number of quenching cycles, leading to the initiation and extension of longitudinal cracks at the interface. The stress is more concentrated at the center of TBC-2, causing cracks to preferentially develop there, resulting in the initial spallation of the coating that gradually extends towards the edges of the sample [42]. Additionally, the further away from the center of the sample, the lower the quenching stress, which in turn reduces the spallation rate. This explains why the area of spallation in TBC-2 initially grows rapidly after the onset of spallation, but then slows down. The cracks within the YSZ layer develop slowly, ultimately forming the transverse and longitudinal cracks shown in Figure 12c, which trigger the spallation of the YSZ ceramic. Thus, the high thermal expansion coefficient of YSZ helps to mitigate the thermal mismatch stress between the GYYSZ and the bonding layer. The interplay of interlayer cracks, interface cracks, and surface cracks in the GYYSZ/YSZ structure collectively leads to the failure of the coating.

**Figure 13:**
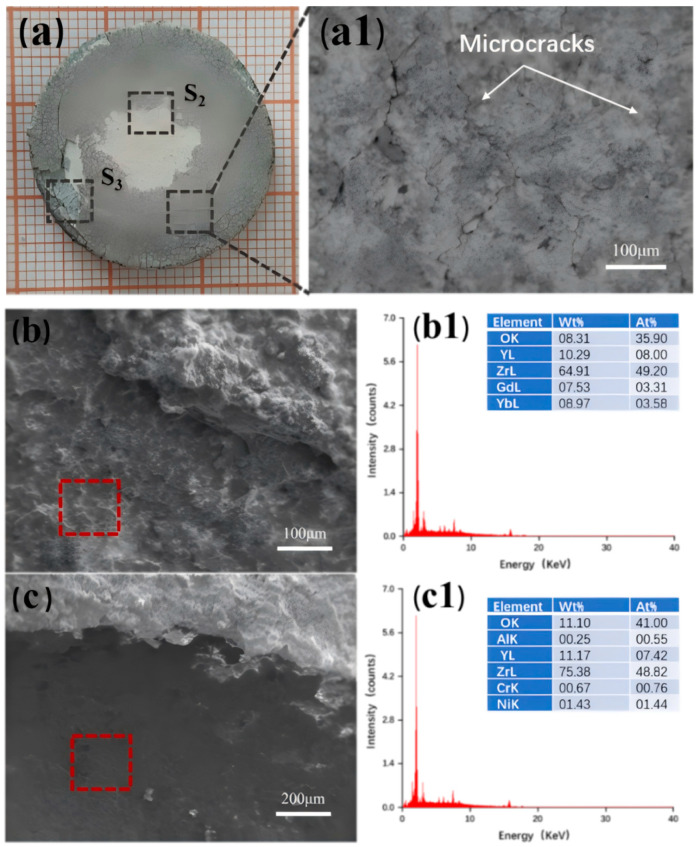
(**a**) The surface morphology of the failed TBC-3 coating; (**a1**) a BSE image of the non-spalled area; (**b**,**b1**) the surface morphology and EDS spectra at positions S2, and at (**c**,**c1**) S3.

Figure 13 shows the failure images of TBC-3. As shown in Figure 13(a1), the unpeeled area exhibits a network of surface cracks, resulting from the release of tensile stress in the thickness direction of the coating. Compared to the surface cracks in TBC-2, the cracks in TBC-3 are finer, which helps delay coating spalling. Compared to the surface cracks in TBC-2, the cracks in TBC-3 are finer, which helps delay coating spalling. Analyzing the different failure characteristics at locations S2 and S3, as shown in Figure 13b,c, reveals the surface morphology and EDS spectra for selected areas. The results indicate that the failure mode at S2 is similar to that of TBC-2, with the peeling edge still consisting of GYYSZ. In contrast, S3 shows significant height differences, indicating direct debonding at this location, and the presence of Ni, Cr, and Al elements in the spectrum confirms that a failure mode similar to that of TBC-1 occurred here.

**Figure 14:**
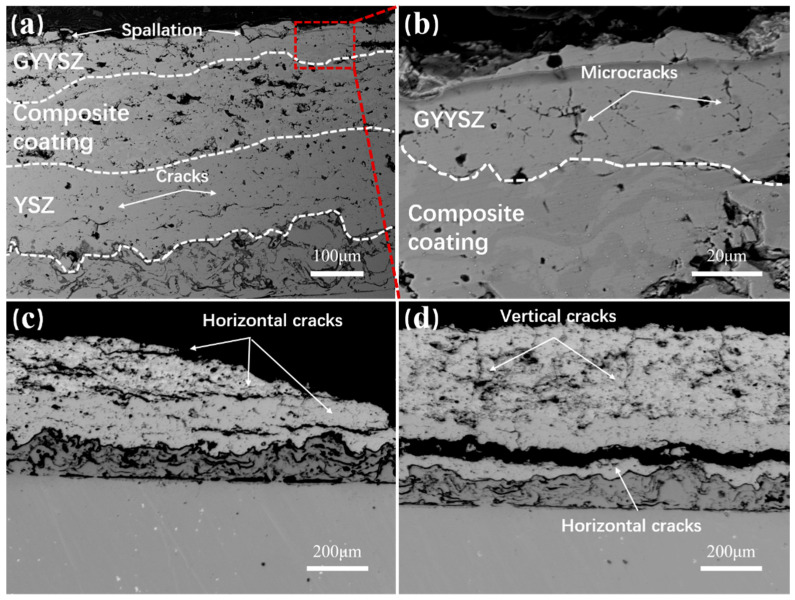
(**a**) The spalled area of TBC-3 after thermal cycling failure; (**b**) a local magnified view; (**c**) the cross-sections of the spall center; the (**d**) edge spall area.

Figure 14 shows cross-sectional images of spalling at the center and edges. In Figure 14a, the thickness of the GYYSZ layer is reduced, and block-like ceramics are observed on the layer, indicating that the spalling occurs within the GYYSZ top layer. There are no obvious longitudinal cracks observed within the composite layer and the YSZ layer, and only minor transverse cracks are present at the interface between NiCrAlY and YSZ. This indicates that optimizing the structure of the thermal barrier coating can significantly reduce the thermal mismatch stress between the components. In the competition between interface cracks and surface cracks, the expansion of surface cracks dominates, leading to the failure and detachment of the coating. In the cross-sectional morphology of the GYYSZ layer, as shown in Figure 14b, numerous microcracks can be observed within the coating. The presence of shorter and higher-density microcracks can reduce the driving force for interface cracks. These microcracks can relieve the thermal mismatch stress at the interface, effectively alleviating interface stress, suppressing the formation and growth of transverse cracks, and thereby delaying coating spallation and enhancing thermal cycling life [43,44,45]. The coating primarily fails through the slow propagation, coalescence, and spallation of cracks. However, as the number of thermal shock cycles increases, interface cracks gradually extend to the state shown in Figure 14b. Figure 14c,d illustrate the cross-sectional microstructure of the spalled TBC-3. During quenching, the thermal gradient stress at the circular edge of the sample is lower. The high-density surface cracks help to relieve the stress within the ceramic layer, allowing the relatively large difference in thermal expansion coefficients at the YSZ/NiCrAlY interface to become the primary driving force for interface crack propagation, resulting in the formation of penetrating transverse cracks at the interface, which represent the main failure mode.

## 4. Conclusions

(1) GYYSZ powder exhibits a single cubic phase of c-ZrO_2_. After undergoing annealing treatments, the GYYSZ coating remains in the cubic phase of c-ZrO_2_. With the rise in annealing temperature, the grain size of the GYYSZ coating also increases, reaching a maximum of 4.66 μm. Additionally, the coating retains a certain porosity during annealing, maintaining approximately 9.9% porosity even at 1500 °C, demonstrating a degree of anti-sintering performance.

(2) By comparing the thermal conductivity and thermal expansion coefficients of the three materials, it is found that GYYSZ has a lower thermal conductivity than YSZ and the composite material, reaching its lowest value of 1.35 W·m^−1^ K^−1^ at 1000 °C, which is 44% lower than that of YSZ. Additionally, GYYSZ has the smallest thermal expansion coefficient, while the thermal expansion coefficient of the composite material falls between that of GYYSZ and YSZ in the temperature range of 100−800 °C. This allows the composite material to serve as a transition layer, alleviating the issue of thermal expansion coefficient mismatch. An analysis of the cross-sections of the three TBCs shows that the spraying thickness is consistent with the design, and EDS results reveal a differential distribution of elements, with irregular boundaries and good bonding performance.

(3) TBC-1 failed after an average of 42.3 thermal shock cycles, resulting in large areas of ceramic layer delamination and a significant drop in quality. The failure was caused by a large thermal mismatch coefficient between the ceramic layer and the bonding layer, leading to concentrated thermal mismatch stress that induced the initiation and rapid propagation of interfacial transverse cracks, ultimately resulting in the formation of through-thickness transverse cracks and the large-scale delamination of the ceramic layer. TBC-2 exhibited a thermal cycling life of 126.3 cycles. The functionally graded coating structure of TBC-3 further optimized the coating design by adding a transition layer, demonstrating excellent thermal shock resistance with an average thermal cycling life of 246.3 cycles, 1.8 times that of TBC-2. The gradient structure effectively reduced thermal mismatch stress, and the fine, dense surface microcracks contributed to a certain degree of toughening. The failure mode of the coating shifted to a mixed mode of block- and point-like delamination.

## Figures and Tables

**Table 1:** Spray parameters for the NiCrAlY bond coat and the TBCs.

Parameters	NiCrAlY Bond Coat	YSZ Coat	GYYSZ Coat	Composite Coat
Current (A)	600	550	600	600
Primary Gas Flow (Ar, L·min^−1^)	46	32	58	58
Secondary Gas Flow (H2, L·min^−1^)	6	8	16	12
Feed Rate (g·min^−1^)	84	64	46	59
Spray Distance (mm)	90	90	70	80
Step Size (mm)	4	2	2	2
Spray Gun Traverse Speed (mm·s^−1^)	1000	1000	1000	1000

**Table 2:** The ionic radii of the four cations in GYYSZ [33].

Ionic	Y^3+^	Gd^3+^	Yb^3+^	Zr^4+^
Ionic radii (nm)	0.102	0.105	0.099	0.084

**Table 3:** The changes in grain size of GYYSZ coatings after heat treatment at different temperatures.

Temperature (°C)	1100	1200	1300	1400	1500
Average grain size (nm)	843	991	1353	1721	4657

**Table 4:** The porosity of GYYSZ coatings in the as-sprayed state and after heat treatment for 5 h.

Temperature (°C)	As-Sprayed	1100	1200	1300	1400	1500
Porosity (%)	21	14.8	13.1	10.5	11.5	9.9

## Data Availability

Data are contained within the article.
